# A virtuous cycle of co‐production: Reflections from a community priority‐setting exercise

**DOI:** 10.1111/hex.13851

**Published:** 2023-08-21

**Authors:** Deborah Ikhile, Devyn Glass, Kat Frere‐Smith, Sam Fraser, Keith Turner, Hasu Ramji, Georgie Gremesty, Elizabeth Ford, Harm van Marwijk

**Affiliations:** ^1^ Department of Primary Care and Public Health Brighton and Sussex Medical School, University of Sussex Brighton UK; ^2^ Academic Health Science Network for Kent, Surrey and Sussex Surrey UK; ^3^ Primary Care and Community Health Services, NIHR ARC KSS (Applied Research Collaboration Kent, Surrey, and Sussex) Surrey UK; ^4^ National Institute for Health and Care Research Applied Research Collaboration for Kent, Surrey and Sussex (NIHR ARC KSS) Surrey UK

**Keywords:** co‐production, shared ownership, shared power, shared responsibilities, shared understanding, shared value, virtuous cycle

## Abstract

**Introduction:**

Co‐production is gaining increasing recognition as a good way of facilitating collaboration among different stakeholders, including members of the public. However, it remains an ambiguous concept as there is no definitive or universal model of co‐production or clarity on what constitutes a good co‐production approach. This paper draws on the reflections of the academic researchers, practitioners and public advisors involved in co‐producing a priority‐setting exercise. The exercise was conducted by the Primary and Community Health Services (PCHS) Theme of the National Institute for Health and Care Research Applied Research Collaboration for Kent, Surrey and Sussex (NIHR ARC KSS).

**Methods:**

We collected data through written and verbal reflections from seven collaborators involved in the PCHS priority‐setting exercise. We used Gibbs' model of reflection to guide the data collection. We then analysed the data through an inductive, reflexive thematic analysis.

**Results:**

A common thread through our reflections was the concept of ‘sharing’. Although co‐production is inherently shared, we used the virtuous cycle to illustrate a sequence of sharing concepts during the research cycle, which provides the underpinnings of positive co‐production outcomes. We identified six themes to denote the iterative process of a shared approach within the virtuous cycle: shared values, shared understanding, shared power, shared responsibilities, shared ownership and positive outcomes.

**Conclusion:**

Our results present a virtuous cycle of co‐production, which furthers the conceptual underpinnings of co‐production. Through our reflections, we propose that positive co‐production outcomes require foundations of shared values and a shared understanding of co‐production as a concept. These foundations facilitate a process of shared power, shared responsibilities and shared ownership. We argue that when these elements are present in a co‐production exercise, there is a greater potential for implementable outcomes in the communities in which the research serves and the empowerment of collaborators involved in the co‐production process.

**Public Members' Contributions:**

Three members of the public who are public advisors in the NIHR ARC KSS were involved in the priority‐setting exercise that informed this paper. The public advisors were involved in the design of the priority‐setting exercise and supported participants' recruitment. They also co‐facilitated the focus groups during data collection and were involved in the data analysis, interpretation and preparation of the priority‐setting report. For this current manuscript, two of them are co‐authors. They provided reflections and contributed to the writing and reviewing of this manuscript.

## 1 BACKGROUND

Co‐production is increasingly gaining popularity as an appropriate way of working with communities and research beneficiaries. Both public involvement and co‐production are largely advocated for by research organisations, policymakers and the healthcare system as two practices that greatly determine the translation of research findings into practice.^1^ The National Institute for Health and Care Research (NIHR) INVOLVE guidance on co‐producing a research project defines co‐production as^2^ ^,p.4^

…an approach in which researchers, practitioners and members of the public work together, sharing power and responsibility from the start to the end of the project, including the generation of knowledge

One of the hallmarks of co‐production is the involvement of nonacademic individuals in different phases of the research process.^3^, ^4^, ^5^ In line with the NIHR's definition, in this paper, we use co‐production explicitly as a collaborative process of research, one that is distinct from public involvement or other forms of working with members of the public.

It is also worth noting that not all cited works of co‐production follow the same process of involving the public along the NIHR guidelines, so, co‐production remains a somewhat elusive or perhaps contested concept fraught with ambiguities.^1^, ^6^, ^7^ In particular, there is little procedural guidance or identification of mechanisms that constitute effective co‐production and how this translates to quality outcomes in practice.^3^, ^4^ There are also several ways of working with stakeholders in research including patient and public involvement (PPI), patient partnership, co‐design, co‐creation and public engagement.^3^, ^8^, ^9^, ^10^ These different ways of working in research further complicate the understanding of co‐production and have resulted in it sometimes being used interchangeably to mean PPI, co‐creation or co‐design.^8^, ^9^, ^11^ While there is evidence as well as increasing interest in trying different ways of working with public members in research, there is limited evidence of what underpins a good co‐production activity and the impacts of co‐production on stakeholders who are not patients or members of the public are not well documented.^3^, ^12^

One way to better understand the elements that contribute to successful collaboration in research activities has been to gather reflections from participants from co‐produced projects. For instance, Tanay et al.^9^ showed the perceptions of participants around their changing roles in co‐produced projects. While there is also a growing body of literature showing the experiences of collaborators in co‐produced research and highlighting the facilitators and barriers to effective collaboration,^10^, ^13^, ^14^, ^15^ more reflection on the process of co‐production will inform best practice and how it is applied in research. Therefore, through reflective accounts of academic researchers, practitioners and public advisors who were involved in coproducing a community priority‐setting exercise, we aimed to (1) further an understanding of how to conceptualise co‐production, (2) show how co‐production can translate into potentially implementable research outcomes and (3) highlight the impacts of co‐production on collaborators.

### 1.1 The primary and community health services community priority‐setting exercise

Primary and Community Health Services (PCHS) is one of the eight themes of the NIHR Applied Research Collaboration for Kent, Surrey and Sussex (ARC KSS). The overall ARC objectives are of providing and evaluating (improvements in) sustainable and integrated regional care^16^: (a) to stimulate the growth of regional applied research capacity, (b) to facilitate and fund end‐of‐pipeline implementation research and (c) to conduct co‐produced (methodological) studies. As the PCHS Theme contributes to these objectives through application to both primary care and community services, we conducted a community priority exercise between 2021 and 2022, using co‐production and public involvement approaches.

The public involvement comprised members of the public and representatives of community organisations who participated in focus group discussions to provide insights on what the research priorities ought to be for the PCHS Theme (Box 1). While the focus of this paper is not on the findings of the priority‐setting exercise, we provide a summary in Box 1.

Box 1 **Summary of the PCHS priority‐setting exercise**

*Purpose*: To refine the research priorities of the PCHS Theme, and to ensure they are defined by local communities.

*Date*: August 2021 to August 2022

*Setting*: Kent, Surrey and Sussex

*Approach*: Co‐production and public involvement.

**Figure 1:**
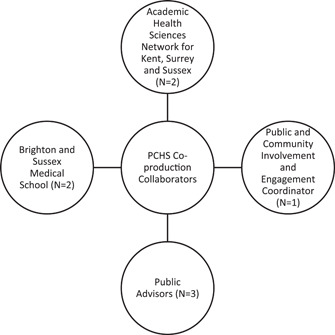
Collaborators involved in coproducing the PCHS priority‐setting exercise. PCHS, Primary and Community Health Services.

- Co‐production: Eight collaborators as outlined in Figure 1. The responsibilities of collaborators during co‐production are outlined in Table 1.
- Public involvement: 22 participants (5 from Kent, 6 from Surrey and 11 from Sussex) were involved through online focus group discussions via Zoom. Participants were recruited widely through adverts shared with local organisations, word of mouth and snowballing. The inclusion criteria for involving participants was that they had to be at least 18 years old and resident in Kent, Surrey or Sussex. The participants had an age range of 18–75 years. Sex distribution was 5 males, 11 females; 6 participants did not disclose their sex.

*Findings*: [1] Improving the ‘front door’ to the NHS, [2] addressing problems in the healthcare system, [3] developing person‐centred care and [4] focus on seldom‐heard groups (e.g., carers, refugees and asylum seekers).

The co‐production element involved eight collaborators who are involved in overseeing and supporting research activities of the PCHS Theme (Figure 1).

The two academic researchers (D. I., D. G.) are employed by Brighton and Sussex Medical School and they coordinate research activities within the PCHS Theme. The academic researchers work closely with the Academic Health Science Network (AHSN) to provide dissemination and implementation support. Two representatives from the AHSN (S. F., G. G.), an implementation manager and an implementation assistant were therefore involved in the exercise, along with a Public and Community Involvement and Engagement (PCIE) coordinator working for the ARC KSS (K. F.‐S.) and three public advisors. For the public advisors, we collaborated with the two advisors attached to the PCHS Theme (K. T., H. R.) and we extended an invitation to public advisors working with other themes of the ARC KSS. Our reason for inviting other public advisors was to ensure we capture as many community views as possible. Only one public advisor from the Social Care Theme was available to collaborate with us at the time. The public advisor was no longer available after we completed the studies and declined the invitation to participate in this publication.

Public advisors are recruited by the ARC KSS through an expression of interest and selection process, and they are attached to each theme to support governance and ensure the theme's activities align with the needs of the local communities. The NIHR ARC for KSS selects public advisors based on their knowledge of their local community, lived experience of health and social care and the extent to which they are embedded into their communities, for example, through their links to local organisations. Public advisors are usually taken through an orientation process by the PCIE Theme of ARC KSS to enable them to perform their roles effectively. K. T. is a community‐based massage therapist in Sussex and he was recruited as a public advisor in 2020. H. R. has over 21 years' experience of working with community and voluntary organisations in Surrey and he was recruited in 2021 just before the priority setting was conceptualised. Both public advisors have links with community and voluntary organisations focusing on mental health, dementia, carers, young people and family support. K. T. and H. R. have established rapport with other members of the PCHS Theme as they have been supporting the theme since their recruitment. Public advisors were paid in line with ARC KSS's PCIE guidelines.

### 1.2 The co‐production process

During the co‐production, the team collaborated through Zoom, telephone calls and texts, emails and MS Teams. As the priority‐setting exercise took place during COVID‐19, all interactions were carried out virtually. The co‐production process for the priority setting involved working with collaborators across different phases of the priority setting (Table 1). After the conceptualisation of the study, one of the researchers and a member of the AHSN worked closely with the PCIE coordinator to advise on the co‐production with public advisors. Public advisors were then involved in the project design, recruitment, data collection, analysis and interpretation and reporting. They refined the research questions, which were initially drafted by one of the researchers, co‐facilitated focus groups along with other collaborators, supported the refining of themes and interpretation during the thematic analysis, and they reviewed and commented on the final report. Due to their extensive knowledge and lived experience of health and social care services in KSS, the public advisors were able to contribute to the priority setting in a way that made the data from the focus group discussions more contextually relevant and grounded in the primary care needs of the local communities. Transcription of the focus group discussions was outsourced to a professional organisation.

**Table 1:** Mapping the roles of the different collaborators during co‐production of the PCHS priority setting.

	D. I.	S. F.	K. F‐S.	K. T., H. R.	G. G.	D. G.
Conceptualisation	❖	❖				
Inception	❖	❖	❖			
Project design	❖	❖	❖	❖		
Recruitment	❖	❖	❖	❖		
Data collection	❖	❖	❖	❖		
Data analysis		❖	❖	❖	❖	❖
Reporting	❖	❖	❖	❖	❖	❖

Abbreviation: PCHS, Primary and Community Health Services.

## 2 METHODS

**Figure 2:**
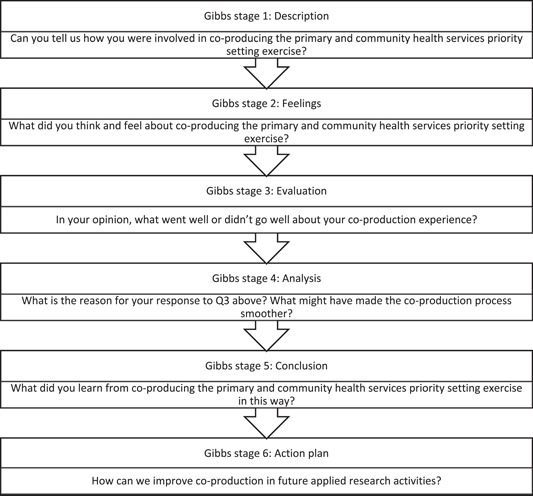
Reflections questions based on Gibbs^20^ reflective cycle.

This paper is based on the reflections of collaborators who co‐produced the PCHS community priority‐setting exercise. Reflection and reflective practice are well embedded in healthcare education and practice^17^ as a way of providing a ‘first‐person narrative that explores personal experience or perspectives on events or issues’.^18^ ^,p.1^ The NIHR ARCs provide research infrastructure to facilitate applied research, that is, research that can be translated into practice to meet the needs of local communities.^19^ To support the reflective process, we used Gibbs^20^ cyclical model of reflection, a practice‐based model to provide a structured and systematic process to reflect on our experiences in the PCHS co‐production process (see Figure 2). There are several models of reflection, but Gibbs' model is popular in academic and healthcare settings to guide reflective writing.^18^, ^21^

### 2.1 Data collection

The reflections were provided by seven out of the eight collaborators involved in the priority‐setting exercise. One of the collaborators declined to participate based on other commitments. The lead researcher, D. I. prepared guiding questions based on the Gibbs model of reflection (Figure 1). Collaborators provided written reflections or shared their experiences via a Zoom call with D. I., who transcribed and collated the conversations. Three collaborators provided written reflections and three offered verbal reflections via video call. D. I. transcribed the verbal reflections and the respective collaborators reviewed their transcripts for accuracy and validation. To avoid unconscious bias or influence on responses, D. I. responded to the questions first before sending to the other collaborators.

### 2.2 Data analysis

We conducted an inductive reflexive thematic analysis using Braun and Clarke's six‐step approach.^22^ Although we used the Gibbs model to guide the data collection, it did not guide our analytical process. Rather, we conducted an inductive analysis to enable the collaborators to draw out their meanings from the qualitative data. This enabled us to maintain the subjectivities of each reflexive account, while embedding the process of reflections in our interpretations of the data.

The analysis was conducted primarily by D. I. with substantial inputs from K. F.‐S. and S. F. D. I. first collated the transcripts into one document and read through the collated transcripts independently for initial coding and to familiarise herself with the data. She then uploaded the transcripts on a shared drive for K. F.‐S., S. F. and D. G. to read through. D. I., K. F.‐S. and S. F. met as a group and collaboratively generated the codes. Afterwards, D. I. synthesised the codes and generated the initial themes which were shared with the other collaborators for their review and comments. Their comments helped develop ‘a richer more nuanced reading’ of the reflections.^22^ ^,p.594^ The defining and naming of the themes were also done collaboratively with inputs from D. I., K. F.‐S., S. F., H. R., K. T. and D. G. All the authors reviewed and contributed to the final results. By analysing the data inductively, we were able to identify the interlinks between the themes, which led us to present our findings using a virtuous cycle.

## 3 FINDINGS

We identified six themes from our analysis, five of which pertained to the notion of sharing: shared values, shared understanding, shared power, shared responsibilities, shared ownership, and positive outcomes. During the data analysis, we recognised the interlink between the themes, as a result, we used the virtuous cycle of continuous improvement to denote an iterative process of a shared approach. A virtuous cycle is a ‘recurring cycle of steps where each step/cycle can reinforce the previous [or next] one in a positive feedback loop of continuous improvement’.^23^ ^,p.613^

### 3.1 Shared values

The first theme we identified was that of shared values. From our reflections, we realised that the starting point of our co‐production process was the identification of a clear purpose for the priority‐setting exercise and why this should be co‐produced. One of the researchers and AHSN implementation manager reflected on how the PCHS Theme was operating within a different primary and community health services landscape as a result of the COVID‐19 pandemic. They explained how this changing landscape spurred the need to interact with local communities to define the ARC's primary care research priorities. The reflection shows that the recognition of the shifting landscape and the need to work with communities were shared among the collaborators during the conceptualisation of the project. It was therefore easy to get their buy‐in on the project.

…we needed to seek a bit more granularity on where we were kind of going with our research as a Theme given that the Primary and Community Health Services Theme is a very broad Theme which does have some kind of key objectives but again, they're quite broad and also, we were trying to conduct research against a completely different landscape… trying to get into communities particularly that are often hard to you know are seldom heard because those really are the ones who have quieter voices and often you know are dismissed or their opinions are diminished. (S. F.)

### 3.2 Shared understanding

The collaborators' reflections demonstrated the need for a shared understanding of co‐production from the start of the project, in a general sense and as applied to specific research activity. To engender a shared understanding of how the priority‐setting exercise would be coproduced, we took a pragmatic approach that involved guidance from the co‐production and PCIE Themes of the NIHR ARC KSS. With their advice, at the start of our project, we prepared a briefing document to share with collaborators who were public advisors to explain and get their input on their involvement and the process of co‐production.

Also, more should be done to improve public members’ awareness of co‐production so they have better understanding of how to negotiate the interactions with other collaborators. My opinion from this exercise is that public members are generally familiar with being involved in projects as study participants and consulted on project ideas, thus being involved yet distanced from the project. For our study, we were clear from the start what their role would be as collaborators and we produced a briefing document to explain what this meant. I believe this approach empowered the public advisors to actively collaborate with us. (D. I.)

### 3.3 Shared power

Our reflections further highlighted how power was shared among collaborators, although to varying degrees and at different points, depending on the responsibilities of each collaborator. The reflections showed that having a shared understanding of how the priority‐setting exercise should be co‐produced gave the collaborators the power to actively contribute in a way that highlighted their strengths and expertise. Even though one of the collaborators stated: ‘I think co‐production is so much about everyone being able to share power’ (S. F.), our reflections highlighted that power was not equally shared throughout the entire process. Although there was equal power sharing during focus groups where all collaborators acted as co‐facilitators, our reflections showed a system of power shift among the collaborators during the other phases, whereby each collaborator led different aspects of the priority setting. For example, during the definition of the themes, the power shifted to the public advisors. We also observed that the involvement of different academic collaborators in later phases could support a shift of power to the public advisors.

I was able to follow the insights of the public advisors [during the data analysis]…The public advisors helpfully provided a narrative and context to support the themes generated by the research assistant from their experiences of the discussions and as active members of their communities. (D. G.)

We also observed that despite the power sharing, there should be a named person accountable for the project, in our case the academic researchers.

…there is still the need to have a person who would have overall responsibility and be held accountable for the project (D. I.)

### 3.4 Shared responsibilities

All collaborators commented on the element of shared responsibilities in their reflections. They highlighted how they saw co‐production as a collaborative effort and valued the different expertise of each collaborator, particularly public members. We reflected on how sharing responsibilities throughout the priority‐setting exercise by negotiating roles and relationships allowed interdisciplinary working and fostered a connected team.

It was a really good team effort. It says somethings about interdisciplinary team working I think, about the different skills and insight people bring and the value of working in this way…That is one of the things I enjoy about the ‘Theme’ approach in the ARC KSS, the whole Theme can support a project and in varying ways, regardless of their official role. (K. F.‐S.)

Our reflections further revealed that shared responsibilities require reciprocity and drawing on others' strengths. For instance, although public advisors did not have academic research experience, they brought in a wealth of experience as community representatives with diverse and multifaceted backgrounds.

It [the priority setting exercise] was also the first piece of co‐produced research I have been involved with, so it was a learning experience for me personally but it was nice to have so much support. (G. G.)

### 3.5 Shared ownership

From our reflections, we observed that by sharing responsibilities, collaborators were able to own aspects of the priority‐setting in a way that would not have been possible in conventional research. The collaborators expressed various feelings around ownership of the priority‐setting process and outcomes. Generally, collaborators felt they were each equally embedded within the process and expressed ownership of that, for example, one collaborator described being part of the ‘actual mechanism’ (K. T.). There was no sense of personal ownership of the outcomes among collaborators, rather a perception of joint ownership as the outcomes were grounded in communities' needs.

I cannot as a researcher claim ownership of the findings and that is a good thing because those findings reflect the actual needs of the communities we serve. (D. I.)

However, one collaborator expressed that they did not feel a sense of ownership towards the outcome of the priority‐setting exercise.

…But that's fine because what I've learned when being involved in various activities is not to become attached to the outcome. I leave it to the Research Lead, as the team may have gathered other views as well. Often, I don't have time to go back and check anyway unless invited by the Research Team to do so…I find it better not to become too attached and instead leave it to their best judgment and move on to the next assignment. So that's the way, I've continued working with ARC. (H. R.)

### 3.6 Positive outcomes

From our reflections, we observed that the co‐produced priority‐setting exercise resulted in the generation of evidence that was aligned with local communities' needs and realities, and had greater potential to be implemented. For instance, one of the priorities we identified was to focus on the needs of informal carers. This priority was also identified through a separate priority‐setting exercise conducted by the Social Care Theme of ARC KSS. This has led to the establishment of a working group focused on co‐designing project(s) to assess and address the needs of informal carers in KSS.

The process of reviewing the themes with the Public Advisors and their input into the development of the outputs enhanced the quality of the outputs and the project more generally…The priority‐setting exercise has also sparked a collaboration with other Themes [of ARC KSS] and has led to the conceptualisation of projects that align with the priorities identified for PCHS as well as other fields, such as Social Care. For instance, a cross‐Theme working group has been formed to initiate projects relating to the needs of carers. (D. G.)

We also identified the impacts of the co‐production process on public advisors. The public advisors felt the co‐production exercise sparked their creativity, gave them a voice, and reinforced their role as boundary spanners, that is the link between the ARC and local communities;

So, there are multiple benefits of co‐production rather than seeing it as a standalone exercise of just collecting information from members of the public. By getting the members of the public in a co‐production exercise, you get their creative juices flowing i.e., their grey cells being more actively engaged in a joint endeavour. (H. R.)

It gives us a voice really and I think that's very important for the community to have voice and to actually feedback and have some ability to help shape it actually because we live in a community we know… I think that's really good that we can be the ‘go‐between’ and help guide the research…I did feel we were listened to, very much so, it was really good… otherwise, you are not really in contact, you just feel that things are done to you or things are asked from you, but you never really hear any more about it and that's it. (K. T.)

### 3.7 The virtuous cycle of co‐production

**Figure 3:**
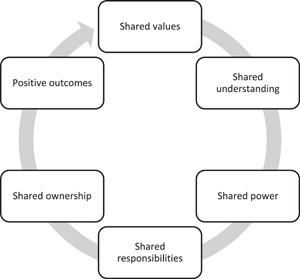
The virtuous cycle of co‐production.

We emphasise the interlink between the themes derived from our reflections on the community priority‐setting exercise and propose a virtuous cycle of co‐production. As mentioned, a virtuous cycle illustrates a sequence of elements that facilitate desirable outcomes. The six themes we identified denote elements of an iterative process of sharing within a virtuous cycle (Figure 3). The first two elements (shared values and shared understanding) occur during the conceptualisation phase of a co‐production activity. Conceiving a project with shared values means collaborators are working with a shared sense of purpose. An agreed and shared understanding of co‐production as concept, and as relates to the project, ensures collaborators hold a transparent set of expectations about the project activity, their roles, responsibilities, and ownership.

We suggest foundations of shared values and shared understanding can facilitate co‐production activity that mobilises the skills and unique contributions of all collaborators. A transparent understanding of the co‐production process can empower active and equal contribution from different collaborators (shared power), and in turn, allows for sharing of responsibilities with consideration for the strengths of individual collaborators. A shared sense of power and responsibility can foster equitable feelings of ownership and influence in the project activity and outcomes. We suggest an iterative process of sharing within this virtuous cycle can empower community collaborators and give rise to research outcomes that have greater potential to be implemented in the communities that the research serves.

We note that in our community priority‐setting exercise, some collaborators did not feel an ongoing sense of ownership over the project. However, they stated they felt influence over the project outcomes as an active and valued member of the team.

## 4 DISCUSSION

The current study analysed the reflections of seven collaborators involved in a co‐produced community priority‐setting exercise. We presented the resulting six themes as a virtuous cycle of co‐production, which furthers our understanding of the conceptual underpinnings of sound co‐production activity. We argue that shared values and a transparent understanding of the application of co‐production provide the necessary foundations for co‐produced research. These foundations facilitate the process of shared power, shared responsibilities and shared ownership. When these elements are present, co‐produced research has the potential to produce research outcomes that represent the needs of communities and are more likely to be implemented. Within the virtuous cycle, we emphasise the importance of sharing power, responsibilities and ownership across all collaborators, which can empower community members and ensure their voices are represented and heard.

We recognise that what can be perceived as the ambiguity of co‐production can make it a difficult concept to grasp, and there is no ‘the’ or correct co‐production approach.^1^, ^5^ Co‐production is largely driven by principles which vary from one research to another and reflects more of a process and a culture.^24^, ^25^ Different approaches have been promoted to further the understanding of co‐production. For instance, Oliver et al. suggested a ‘cautious approach’ to co‐production to maximise its benefits over the costs.^3^ A common principle that is inherent in co‐produced research is that of sharing.^1^, ^9^ The INVOLVE guidance published in 2018 highlight the sharing of power and responsibilities.^2^ Through our reflections we show that co‐production additionally requires underlying shared values, shared understanding and can benefit from a shared ownership process.

The first element of our proposed virtuous cycle is shared values. Shared values are another ambiguous concept closely linked to various disciplines.^26^ However, underlying shared values have been identified as an incentive for community involvement in co‐production.^27^, ^28^ The incentive in the case of our priority setting was doing research in a different way: from the ground up by working with local communities through co‐production and public involvement. All collaborators were affiliated with the NIHR ARC KSS and valued bottom‐up research, which motivated them to coproduce the priority‐setting exercise with the PCHS Theme. Therefore, we surmise that an affiliation with research infrastructures such as the NIHR ARCs would promote shared values in co‐producing applied health and social care research.

Co‐production exercises often do not follow a definitive model, but some frameworks have been introduced to foster collaborative or participatory research, for example, Experience Based Co‐Design and Always Events®. Although these frameworks are pertinent to health service development and quality improvement,^6^, ^29^, ^30^ existing studies recommend that it is crucial to adapt the co‐production process to the needs of the research and collaborators.^9^, ^31^ This requires defining the parameters and having a shared understanding of the co‐production process at the start of the project. In agreement with existing evidence, having a shared understanding of the co‐production process is one of the determinants of success in co‐produced projects.^9^ This is reflected in our proposed virtuous cycle. In the priority‐setting exercise, we reflected that a shared understanding nurtured collaboration in a way that allowed for seamless (even unspoken) negotiation of power.

Although sharing power has been identified as a key principle of co‐production, a truly equal distribution of power in co‐production exercises is challenging as there is no clarity on how this can be achieved.^4^, ^31^ Power is also one of the drivers of tension and interpersonal conflicts among collaborators in co‐produced research.^1^, ^9^, ^27^ Power differentials among collaborators can give rise to inequalities if not properly managed.^2^ One way to ensure issues related to power do not become a hindrance is through power shifting. With our reflections, we demonstrated that a power shifting approach was used in our priority‐setting exercise. This is consistent with some existing evidence that has conceptualised sharing power.^2^, ^3^ However, this is contrary to other evidence, which portrays power shifting as uni‐directional from researchers to members of the public,^3^, ^32^, ^33^ we show that this power shift is bidirectional and can also occur from members of the public to the researchers. For instance, during the data analysis and reporting, the public advisors' embedded knowledge of their local communities guided how the final themes were named and the presentation of the priority‐setting findings. In this regard, their power as members of the communities enabled the researchers to present the findings in a practical and contextually appropriate manner.

Another way of mitigating power tensions is through a transparent definition of roles and responsibilities.^27^ The use of a briefing document at the start of our priority‐setting facilitated a shared understanding of collaborators' expectations and roles. We see this in the virtuous cycle, where a sound basis of shared understanding contributes to shared power. Using briefing documents in co‐production can aid the process of shared understanding and aligns with Hickey et al.'s guidance. They recommend ‘establishing ground rules' at the start of a co‐production activity.^2^ ^,p.9^ Another approach is through reflexivity on how power is being shared through the co‐production process.^4^, ^11^, ^32^ This sort of reflexivity can only be achieved by a mutual understanding of each collaborator as a project asset, with the voice and opportunity to influence the course of the research process. The notion of power as the ability to influence resonates with existing evidence.^34^

The greater the power wielded by collaborators, the greater their sense of responsibility. Responsibilities can only be effectively shared where there is diversity of roles, strengths and experiences. Expectations and roles need to be clear and well communicated to ensure that all collaborators can carry out their roles effectively. Although we used a briefing document to delineate roles, another good way to facilitate transparency in roles and responsibilities is to use an online shared platform for open and continuous communication.^35^ This opportunity for continuous communication would also foster continuity and enable increased opportunities for shared responsibilities. Future co‐production activities should ensure that they are considered and where practicable have a shared platform to aid open communication, transparency, and shared responsibilities.

The last aspect of sharing identified from our reflections is shared ownership. The ownership of the research data or findings is a crucial consideration in qualitative research.^36^ This is even more pertinent for applied research where the focus is on research that can be translated into actions. Shared power and shared responsibilities during the process do not automatically translate into a sense of ownership as we found out from our reflections, where one of the collaborators described handing back ownership of the research outcomes, perceiving the researchers as integral in drawing together the final results. We recognise that the collaborator had been previously invited to participate to varying degrees across different projects, thus feeling that the ownership of the outcomes rested with the lead researcher. Additionally, this may in part be explained by a perception that competencies relating to research methodology and evaluation gave the overall power and ownership to the professionals. For instance, Tanay et al.^9^ highlighted that all participants in a co‐designed project felt that they were co‐owners except the patient participants. This highlights the potential reticence of collaborators who do not consider themselves as ‘professionals’ to lay claim to, what they believe is, a professional piece of work. During co‐production, more work needs to be done to ensure all collaborators, especially public advisors and other members of the public, feel that they own the outcomes and outputs.

Research projects are co‐produced for several reasons, including the potential for improved quality of research findings and the increased possibility of meaningful impact.^3^ According to Hickey^1,p.1^, the history of co‐production is associated with the ‘design and improvement of services’ and being able to respond to the realities of communities. For applied research infrastructures such as the ARCs, co‐production is a core mechanism of facilitating research that has a greater potential of being implemented to meet the needs of local communities. This paper is pertinent for research infrastructures as it provides a model for developing co‐production activities that can maximise the potential for translation of knowledge into practice.^24^ In the case of our co‐produced priority setting, we identified the needs of informal carers as a priority in Kent, Surrey and Sussex. Although we recognise that this finding may have been identified through conventional research methods, through co‐producing with the public advisors, the significance and extent of issues surrounding the needs of informal carers were accentuated. Our co‐production process further facilitated the translation of knowledge into practice by providing a strong rationale for establishing a working group and designing projects to address the needs of informal carers in the region.

We also highlight the empowerment of public advisors as an impact of co‐production. Empowering public advisors amplified their roles as community‐based boundary spanners, that is individuals who live in local communities in which they serve and facilitate the flow of information between the communities and service providers.^37^ A recently published scoping review revealed that community‐based boundary spanners (such as navigators, peer researchers, community health workers, lay workers) play a role critical for bridging the gap between academics and communities, and fostering access to seldom heard groups.^37^ Public advisors who collaborated in the co‐production process play a similar role. For instance, one of them described himself as a ‘go‐between’ and explained how he acts as a boundary spanner by providing feedback on the needs of communities to the researchers in a way that helps shape the research process and focus within the ARC.

## 5 LIMITATIONS

The transferability of our findings may be limited as the perspectives presented in this paper draw on a regionally co‐produced project (Kent, Surrey and Sussex). Also, the priority setting was community focused and we did not collaborate with primary and community care providers. We further realise that the extent to which the public advisors act as boundary spanners may be restricted by the number, location and type of local organisations they have links to. For instance, the two public advisors involved in our co‐production exercise were from Surrey and Sussex, there was no collaborator from Kent. These limitations emphasise the need for a shared understanding of the context (types of collaborators, settings etc.) of the co‐produced research project.

## 6 CONCLUSIONS

Embedding co‐production in research is regarded as best practice that facilitates a process of collaboration among different stakeholders and can greatly determine the quality of research findings. Despite the growing shift towards co‐production as a way of creating impactful research, there remains limited evidence on the conceptual underpinnings of successful co‐production. Drawing on reflections from collaborators of a recent priority‐setting exercise conducted within ARC KSS, we contribute three additions to co‐production research. First, we propose a virtuous cycle of co‐production, which presents an iterative process of sharing as one way of conceptualising co‐production. The virtuous cycle demonstrates that the principles of co‐production go beyond sharing power and responsibility. There is also a need for shared values, shared understanding and shared ownership of the process and outcomes. Second, we showed that our co‐production exercise resulted in research outcomes that may have more potential to be implemented given their alignment with the public advisors and their local communities. Third, we show how the co‐production process empowers public advisors as boundary spanners between research and local communities. This paper provides a new model of co‐production, which can guide applied health and care researchers in co‐production activities and help maximise positive research outcomes.

## AUTHOR CONTRIBUTIONS

Deborah Ikhile conceptualised the idea for this manuscript and designed the data collection guide and led the data analysis with contributions from other authors. Deborah Ikhile, Sam Fraser, Devyn Glass, Kat Frere‐Smith, Hasu Ramji, Keith Turner and Georgie Gremesty provided reflections that informed the manuscript. All authors contributed to the first draft of the manuscript, reviewed, edited and approved the final manuscript.

## CONFLICT OF INTEREST STATEMENT

The authors declare no conflict of interest.

## Data Availability

Research data are not shared due to privacy and confidentiality.
